# Long non-coding RNA RPAIN regulates the invasion and apoptosis of trophoblast cell lines via complement protein C1q

**DOI:** 10.18632/oncotarget.13826

**Published:** 2016-12-09

**Authors:** Xuejing Song, Can Rui, Li Meng, Rui Zhang, Rong Shen, Hongjuan Ding, Jun Li, Jingyun Li, Wei Long

**Affiliations:** ^1^ Department of Obstetrics, Obstetrics and Gynecology Hospital Affiliated to Nanjing Medical University, Nanjing, China; ^2^ Fourth Clinical Medicine College, Nanjing Medical University, Nanjing, China; ^3^ Nanjing Maternity and Child Health Medical Institute, Obstetrics and Gynecology Hospital Affiliated to Nanjing Medical University, Nanjing, China; ^4^ State key Laboratory of Reproductive Medicine, Department of Plastic and Cosmetic Surgery, Maternal and Child Health Medical Institute, Obstetrics and Gynecology Hospital Affiliated to Nanjing Medical University, Nanjing, China

**Keywords:** lncRNA, RPAIN, early onset preeclampsia, complement protein C1q

## Abstract

Long non-coding RNAs (lncRNAs) are key regulatory molecules that are involved in a variety of biological processes and human diseases. Their impact on early onset preeclampsia remains unclear. In this study, we tested the expression of RPAIN (transcript variant 12 of RPA interacting protein, a non-coding RNA, NR_027683.1) in placenta tissues derived from 25 pregnant women with PE and 15 healthy pregnant women using quantitative real-time PCR. The effect of RPAIN on trophoblast proliferation, invasion, and apoptosis and the underlying mechanisms were examined in trophoblast cell lines (HTR-8/SVneo). The results showed that RPAIN expression levels were significantly increased in early onset preeclamptic placentas compared to normal controls. The proliferation and invasive abilities of the trophoblast cells were significantly inhibited, and the apoptosis abilities of the trophoblast cells were significantly promoted when RPAIN was overexpressed. In addition, the overexpression of RPAIN inhibited the expression of complement protein C1q. Furthermore, C1q overexpression rescued the decreased cell invasion and enhanced cell apoptosis in RPAIN-overexpressing trophoblast cells. Our results suggest that increased RPAIN levels may contribute to the development of preeclampsia through regulating trophoblast invasion and apoptosis via C1q. Therefore, we proposed RPAIN as a novel lncRNA molecule, which might contribute to the development of PE (preeclampsia) and might compose a potential diagnostic and therapeutic target for this disease.

## INTRODUCTION

Preeclampsia is a pregnancy-specific syndrome that affects 3–5% of pregnancies and is traditionally diagnosed by the combined presentation of high blood pressure and proteinuria [1]. Preeclampsia is one of the main causes of maternal, foetal and neonatal mortality, especially in low-income and middle-income countries [2, 3]. This syndrome is generally defined as the new onset of hypertension in association with proteinuria or multiple organ complications at or after the 20th week of gestation. Several varying assumptions exist regarding the pathogenesis of early onset preeclampsia, such as aberrant immune responses [4], oxidative stress [5], abnormal placental development and function [5], inflammation [6] and genetic factors [7, 8]. The exact aetiology and pathophysiology remain uncertain. Although the primary mechanism of preeclampsia remains unknown, considerable evidence indicates that the preeclampsia placenta is characterized by an abnormal trophoblast invasion, deficient maternal spiral artery modification, and increased apoptosis of trophoblastic cells [9–11].

Complement protein C1q is a soluble innate immune pattern recognizing molecule [12]. C1q also plays important roles in pregnancy. C1q is distributed widely in human decidual stroma and is synthesized by migrating extravillous trophoblasts [13]. Recently, several reports indicated that C1q plays a major role in enhancing trophoblast invasion of decidua and that defective local production of C1q may be related to pregnancy disorders, such as preeclampsia, characterized by poor trophoblast invasion [13–15].

Long non-coding RNAs (LncRNAs) are a class of transcripts whose lengths exceed 200 nt. Initially, lncRNAs were thought to be transcribed noise. However, an increasing number of studies have reported that these lncRNAs have a series of important functions and participate in the development of many diseases [16–19], including cancers, cardiovascular diseases and neurovascular-related disorders. Recently, some lncRNAs have been shown to be associated with preeclampsia [20–22].

Previously, based on a lncRNA microarray analysis, we found that lncRNA RPAIN (transcript variant 13 of RPA interacting protein, a non-coding RNA, NR_027683.1) was overexpressed in preeclampsia placenta tissues. The full length of the RPAIN gene is 1567 bp, and the gene is located on human chromosome 17p13.2. In this study, we aimed to investigate the possible role of lncRNA RPAIN in the pathophysiology of early onset preeclampsia and its function in trophoblast biology.

## RESULTS

### RPAIN is increased in preeclampsia placenta tissues

**Figure 1:**
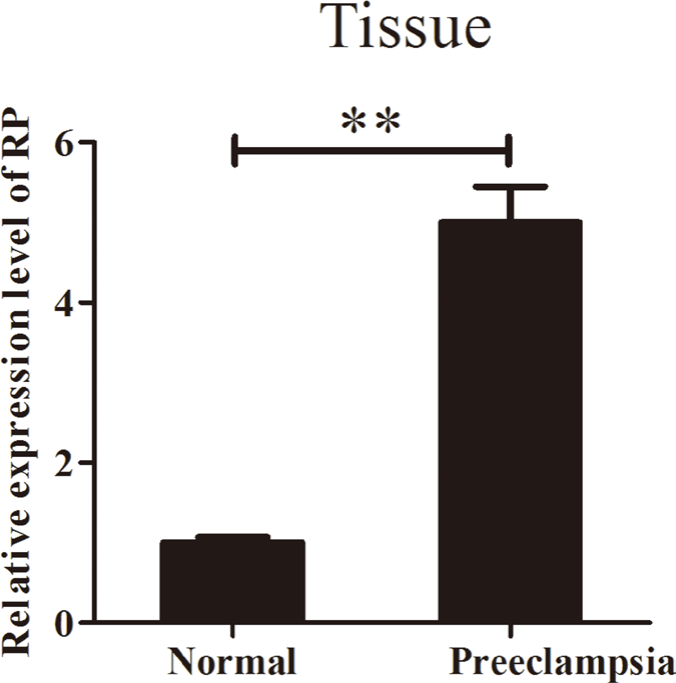
RPAIN expression in preeclampsia placenta tissues and normal placenta tissues RPAIN was overexpressed in preeclampsia placenta tissues. Data are expressed as the mean ± SD. **P < 0.01.

To investigate the potential role of RPAIN in the pathophysiology of PE, we first examined the expression level of RPAIN in 25 preeclampsia placenta tissues and 15 normal tissues using quantitative real-time PCR (qRT-PCR) (*P* < 0.05). The data indicated that RPAIN was upregulated in the early onset preeclampsia samples compared to the normal samples (Figure 1). The study population characteristics are shown in Table 1.

**Table 1:** Clinical characteristics of study patients

Characteristics	Normal pregnancy (*n* = 15)	Preeclampsia (*n* = 25)	*P* value
Maternal age (years)	29.2 + 3.7	30.7 + 5.6	*P* > 0.05
Maternal BMI	26.0 + 2.2	28.9 + 3.8	^*^P < 0.01
SBP (mmHg)	N/A	167.7 + 16.7	^*^P < 0.01
DBP (mmHg)	N/A	108.4 +12.6	^*^P < 0.01
24 h urine protein (g)	N/A	6.0 + 2.9	^*^P < 0.01
Gestational age (week)	33.8 +1.7	33.1 + 1.7	*P* > 0.05
Infant birth weight (g)	2253.4 + 395.8	1624.3 + 290.1	^*^P < 0.01

Abbreviations: Data are presented as the mean ± SD (SD: standard deviation). *P* value, preeclampsia compared with normal pregnancy, ^*^P < 0.01. N/A: Not Applicable.

BMI, body mass index. SBP, Systolic blood pressure. DBP, Diastolic blood pressure.

### Overexpression of RPAIN suppresses the proliferation and invasion of human trophoblast cells (HTR-8/SVneo)

**Figure 2:**
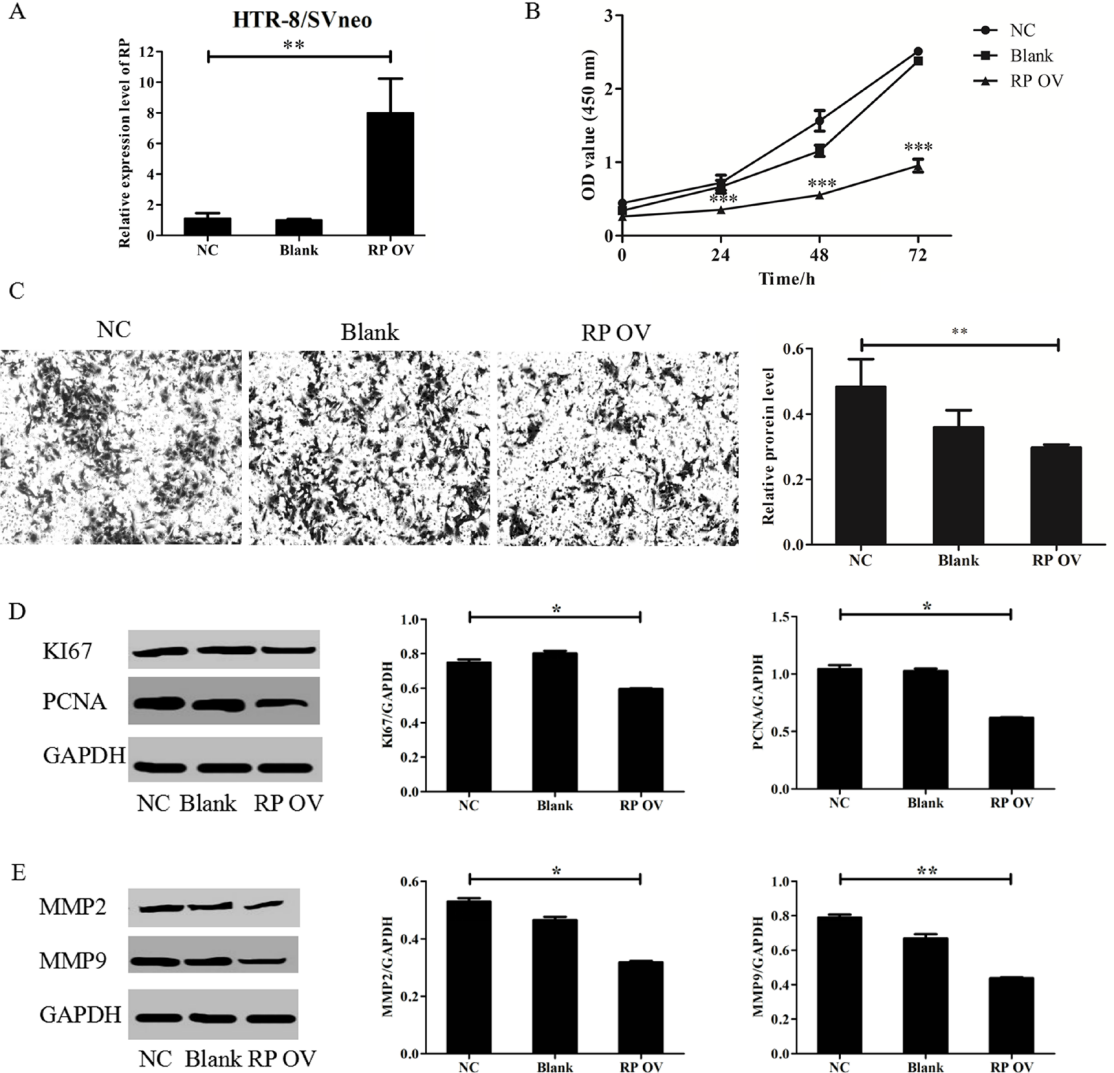
RPAIN suppressed HTR-8/SVneo cells proliferation and invasion (**A**) RPAIN overexpression efficiency was validated by qRT-PCR. Lentivirus infection of RPAIN in HTR-8/SVneo cells upregulated RPAIN expression (approximately 7-fold). (**B**) RPAIN overexpression significantly suppressed HTR-8/SVneo cell proliferation. (**C**) RPAIN overexpression significantly inhibited HTR-8/SVneo cell invasion. (**D**) Western blot and densitometry analysis revealed that KI67 and PCNA protein expression was reduced when RPAIN was overexpressed. (**E**) Western blot and densitometry analysis indicate that MMP2 or MMP9 protein expression was decreased when RPAIN was overexpressed. RP OV: overexpression of RPAIN lncRNA. NC: negative control, overexpression of empty vector. Blank: treated with nothing. *P < 0.05, **P < 0.01, ***P < 0.001, versus NC group.

HTR-8/SVneo cells were transfected with lentiviruses targeting RPAIN after seeding into 6-well plates overnight. The cells were then harvested after 72 hours for RNA extraction. The gene overexpression efficiency was assessed by qRT-PCR. RPAIN exhibited a 7-fold upregulation compared to the negative control (Figure 2A). We then investigated the biological roles of RPAIN in trophoblast cells. We performed a CCK8 proliferation assay and a Transwell invasion analysis to investigate the *in vitro* biological functions of RPAIN. We found that RPAIN upregulation in trophoblast cells significantly inhibited cell proliferation and invasion abilities (Figure 2B, 2C). We further examined several key proliferation and invasion elements in HTR-8/SVneo cells overexpressing RPAIN. Compared to the control cells, PCNA, KI67, MMP2 and MMP9 levels were reduced in cells transfected with RPAIN lentiviruses (Figure 2D, 2E). In general, the above results suggest that increased RPAIN expression led to a sensitization of the proliferative and invasive pathway in the HTR-8/SVneo cells.

### RPAIN promotes human trophoblast cell apoptosis

**Figure 3:**
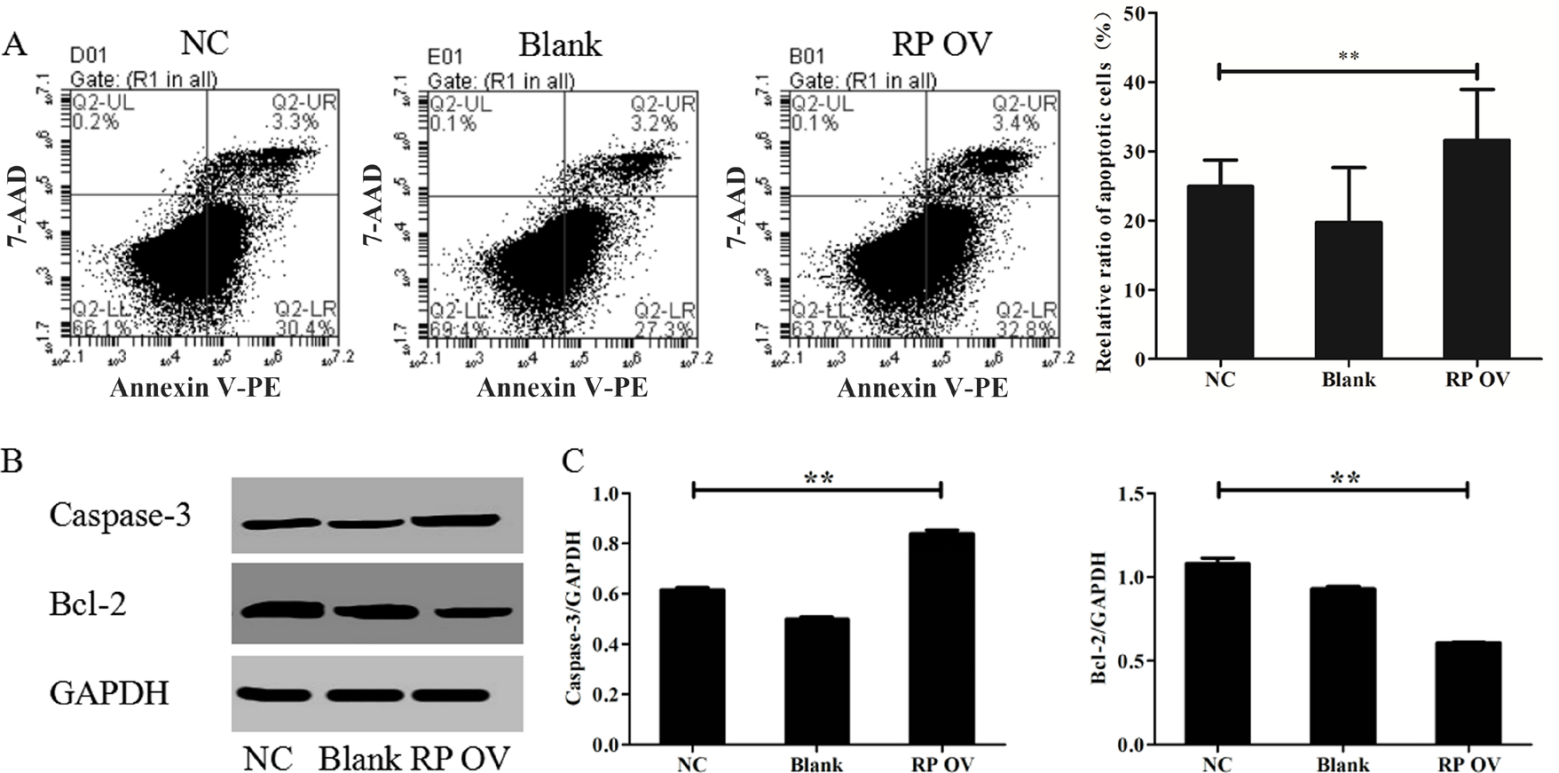
RPAIN promoted HTR-8/SVneo cell apoptosis (**A**) Flow cytometric analysis reflect that the cells transfected with RPAIN lentiviruses showed more apoptotic cells. (**B**) Western blot analysis was used to evaluate the expression of Caspase-3 and Bcl-2. (**C**) Densitometric analysis of Western blot results. RP OV: overexpression of lncRNA RPAIN. NC: negative control, overexpression of empty vector. Blank: treated with nothing. **P < 0.01, versus NC group.

To study the effect of overexpressing RPAIN on HTR-8/SVneo apoptosis, we conducted a flow cytometric analysis. The results reflect that the cells transfected with the RPAIN lentiviruses exhibited more apoptotic cells compared to the control cells. The flow cytometry revealed that RPAIN overexpression expedites apoptosis in HTR-8/SVneo cells (32.8% ± 3.4%, 30.4% ± 3.3%) compared with non-transfected cells (27.3% ± 3.2%) (Figure 3A). Then, we examined the factors associated with apoptosis in the HTR-8/SVneo cells overexpressing RPAIN using Western blot. Compared with the control cells, Caspase-3 levels increased and Bcl-2 levels were reduced in the cells transfected with RPAIN lentiviruses (Figure 3B, 3C).

### RPAIN inhibits expression of C1q

**Figure 4:**
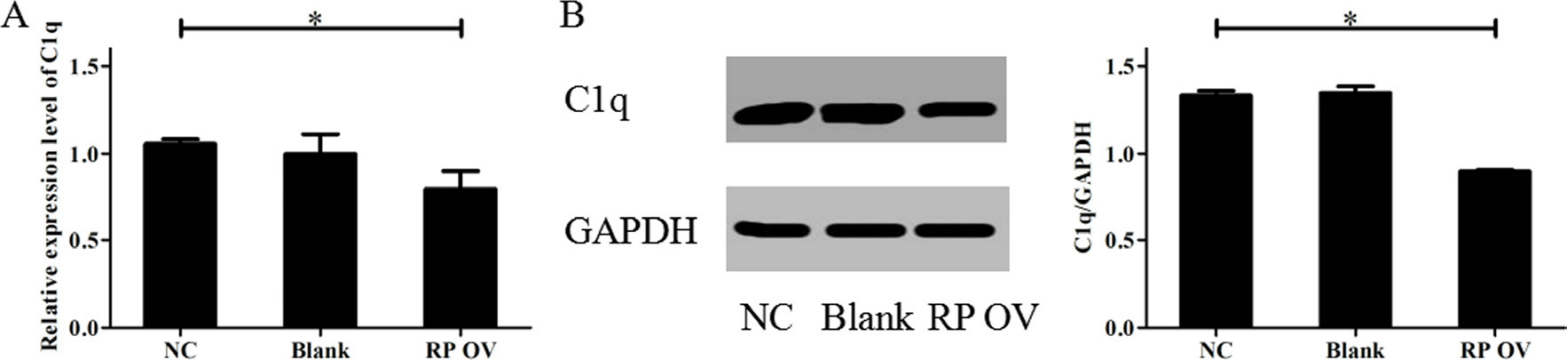
Overexpression of RPAIN inhibited C1q expression (**A**) The qRT-PCR results showed that C1q expression was reduced when RPAIN was overexpressed. (**B**) Western blot results showed that the protein expression of C1q was decreased when RPAIN was overexpressed. RP OV: overexpression of RPAIN lncRNA. NC: negative control, overexpression of empty vector. Blank: treated with nothing. *P < 0.05, versus NC group.

To identify the mechanisms of RPAIN, we adopted UCSC and BLAST to analyse its position. The analysis of the location of RPAIN in the genome revealed that C1q is adjacent to RPAIN. Next, we assessed the effect of RPAIN on C1q levels by performing qRT-PCR. Interestingly, the overexpression of RPAIN in HTR-8/SVneo cells resulted in a significant reduction in C1q expression levels (Figure 4A). Moreover, Western Blot revealed that RPAIN overexpression caused a suppression of C1q in HTR-8/SVneo cells (Figure 4B, 4C). In conclusion, these results suggest that C1q is a potential target of RPAIN in preeclampsia.

### C1q is a functional target gene of RPAIN

**Figure 5:**
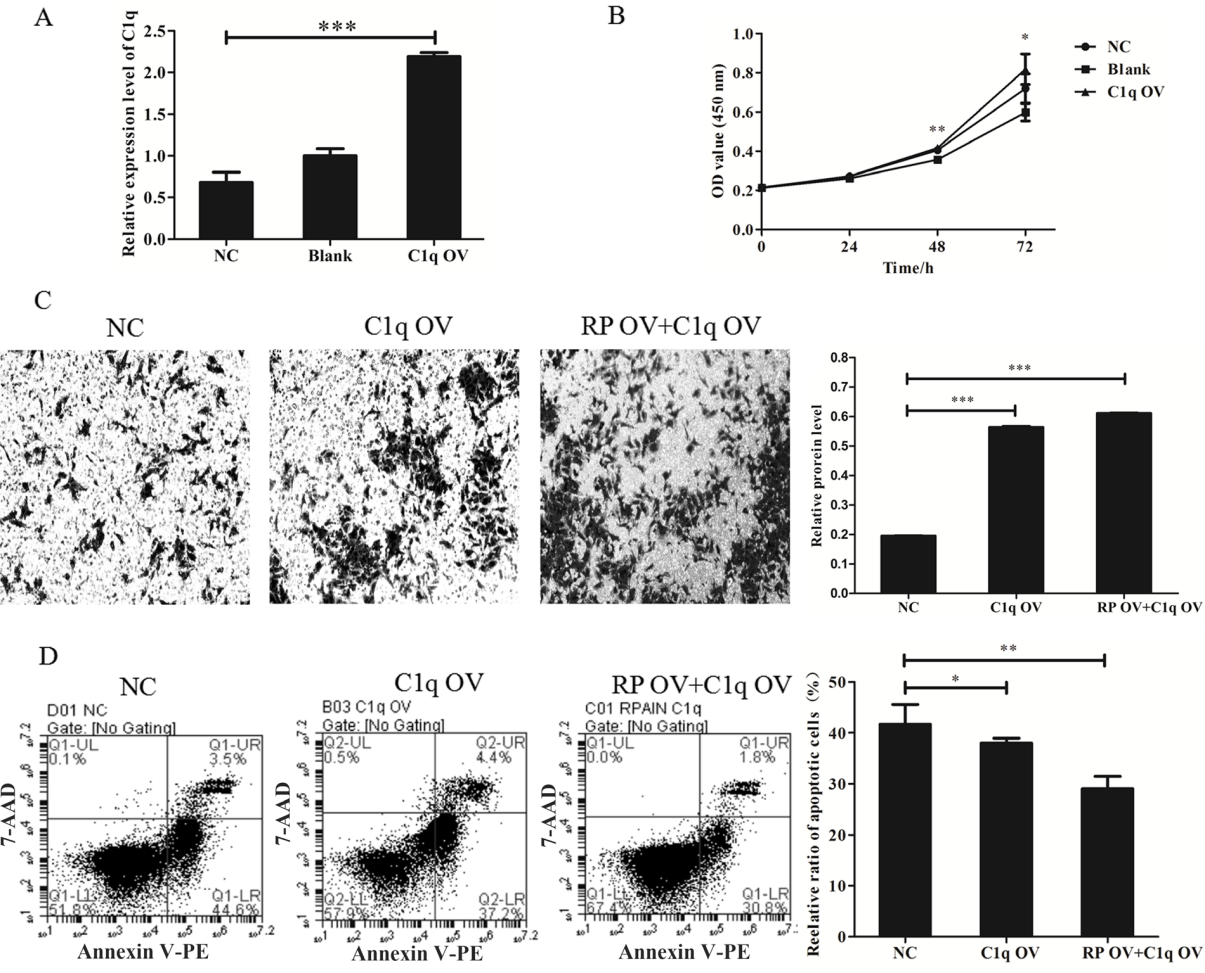
LncRNA RPAIN regulates cell invasion and apoptosis in HTR-8/SVneo cells via C1q-mediated pathway (**A**) The qRT-PCR results showed that lentivirus infection of C1q in HTR-8/SVneo cells upregulated C1q expression (greater than 3-fold) (***P < 0.001). (**B**) CCK-8 assay showed that cell proliferation was promoted when C1q was overexpressed. (**C**) C1q overexpression increases cell invasion of HTR-8/SVneo cells, whereas co-overexpression of RPAIN and C1q restored cell invasion of HTR-8/SVneo cells. (**D**) Flow cytometry analysis revealed that C1q overexpression could decrease cell apoptosis of HTR-8/SVneo cells, whereas co-overexpression of RPAIN and C1q also inhibited apoptosis of HTR-8/SVneo cells. RP OV: overexpression of RPAIN lncRNA. NC: negative control, overexpression of empty vector. C1q OV: overexpression of C1q. RP OV+C1q OV: co-overexpression of RPAIN and C1q. Blank: treated with nothing. *P < 0.05, **P < 0.01, ***P < 0.001, versus NC group.

In previous experiments, RPAIN overexpression caused decreased expression of C1q in HTR-8/SVneo cells. Next, we clarified whether C1q might promote cell proliferation, invasion and apoptosis in contrast to RPAIN overexpression. We found that C1q overexpression (Figure 5A) in HTR-8/SVneo cells increased cell proliferation and invasion abilities and reduced cell apoptosis abilities (Figure 5B–5D), which is in contrast to the effect of RPAIN overexpression. Thereafter, we generated a “rescue” assay to investigate the effect of RPAIN overexpression in the presence of C1q overexpression by lentivirus infection of C1q after stable infection of RPAIN in HTR-8/SVneo cells. The experimental results of invasion and apoptosis indicated that enforced expression of C1q partly restored the invasive and apoptotic abilities of HTR-8/SVneo cells (Figure 5C, 5D). In general, these results indicated that C1q is a functional target gene of RPAIN in preeclampsia.

## DISCUSSION

Accumulating evidence has indicated that lncRNAs participate in preeclampsia. The associations between SPRY4-IT1, maternally expressed gene 3 (MEG3), LOC391533, LOC284100, CEACAMP8 and metastasis-associated lung adenocarcinoma transcript-1 (MALAT-1) with preeclampsia have been reported, indicating that these lncRNAs may be novel modulators in the process of preeclampsia occurrence and progression [20–23]. In this study, we show that lncRNA RPAIN is upregulated in preeclampsia and functions in preeclampsia via the complement protein C1q.

Preeclampsia is a pregnancy-specific disorder. Its pathogenesis is related to a variety of factors, and the placenta is the primary cause of the disease [24]. Dogma states that there is an abnormal trophoblast invasion of the decidual and myometrial spiral arteries in preeclampsia that is related to the pathophysiology [10]. In general, the trophoblasts’ capacity for proliferation and invasion plays a crucial role in successful placental development [25]. Apoptosis is an essential feature of normal placental development; however, the process is also involved in the pathophysiology of PE [26]. Although the apoptosis of trophoblastic cells increases in normal placentas with advancing gestation, increased apoptosis of trophoblastic cells has been observed in pregnancies complicated by PE [27, 28]. Here, we found that lncRNA RPAIN inhibited proliferation, invasion and promoted apoptosis of trophoblast cells HTR-8/SVneo, indicating that lncRNA RPAIN may be one of the causes of PE.

Antisense lncRNAs typically regulate their counterpart sense mRNAs in cis [29]. We found that complement protein C1q was localized adjacent to RPAIN lncRNA. Overexpression of RPAIN reduces C1q expression. Restoring C1q expression reversed the regulation of RPAIN on trophoblast (HTR-8/SVneo) cell invasion and apoptosis. The proximal promoter of C1QBP is under the control of several transcription factors, including SP1, ZNF32 and Tbx1. RPAIN may recruit the transcriptional factors to the C1QBP promoter to inhibit C1QBP expression [30–32]. The functional interactions of lncRNAs, miRNAs and mRNAs could offer a new explanation for the pathogenesis of PE [33]. Using a miRBase sequencing matching strategy (www.mirbase.org/search.shtml) as described in the literature [34], we found that lncRNA RPAIN contains potential binding sites for the following 4 miRNAs: miR-3167, miR-1249-5p, miR-877-5p, and miR-5001-5p. Whether RPAIN interacts with these microRNAs to regulate trophoblast activity requires further exploration. Interestingly, 7 interpartners, including Upf1, JARID2, PABPC1, Celf1, FUS, Tdp43 and Fus/TLS, interact with Mouse Rpain as determined using NPInter v3.0 (www.bioinfo.org/NPInter/) [35]. Whether RPAIN interacts with these proteins to regulate trophoblast activity also requires further exploration.

According to previous research, C1q plays an important role in maintaining the invasive ability of trophoblast cells and spiral artery remodelling [14, 36]. Studies have noted that trophoblasts and cancer cells share similar biological characteristics in their proliferative and invasive characteristics [37, 38]. One study showed that C1q stimulates cell proliferation and migration in breast cancer cells and is also upregulated in human lung and colon cancer cell lines [39]. Therefore, we hypothesized that abnormal RPAIN in the placenta might play a significant role in the pathogenesis of preeclampsia; abnormal expression of RPAIN in preeclamptic placentas could exert an aberrant regulation effect on the invasion and apoptosis of trophoblasts via C1q. C1QBP is a substantial component of the mPTP complex, regulates oxidative phosphorylation and inner mitochondrial membrane permeability [40]. Aberrant regulation of C1QBP is sufficient to induce mPTP opening and subsequent mitochondria-dependent apoptosis [29, 40, 41]. Therefore, RPAIN regulates trophoblast apoptosis through controlling C1QBP expression in a mitochondria-dependent manner.

In addition, it is well known that PCNA/KI67 and Caspase-3/Bcl-2 are important molecular pathways in cancer cell proliferation and apoptosis. Due to the common characteristic invasive behaviour of trophoblast cells and tumour cells, the molecular pathways (MMP2/MMP9) of these two types of cells are strikingly similar. In our study, RPAIN overexpression caused changes in the expression of these classic enzymes and proteins that correspond to the transformation into invasive cells and other biological processes such as proliferation and apoptosis. Overexpression of RPAIN suppresses the proliferation and invasion of human trophoblast cells following 48 h of incubation. The decrease of cell proliferation will inevitably lead to the decrease of the number of invasive cells. However, molecular markers such as KI67 and PCNA (cell proliferation related markers) protein expression were both decreased, and MMP2, MMP9 (cell invasion related markers) also decreased their protein expression. Therefore, both proliferation and invasion of trophoblast cells could be affected by RPAIN overexpression.

Another limitation of our study was the use of the transformed trophoblast cell line HTR-8/SVneo cells. These cells may not replace primary trophoblasts completely. However, we exogenously infected RPAIN lentivirus to evaluate its effects on HTR-8/SVneo cells in our work. To some extent, our study demonstrated that RPAIN may inhibit invasion and promote apoptosis of preeclampsia by downregulating C1q. The present study suggests that the RPAIN/C1q pathway could represent a mechanism of pathogenesis in preeclampsia. And RPAIN might compose a potential diagnostic and therapeutic target for this disease.

## MATERIALS AND METHODS

### Patients and clinical sample collection

Written informed consent was obtained from all patients, and the study was approved by Nanjing Maternal and Child Health Hospital. In total, 25 early onset preeclampsia pregnant women and 15 control patients who received a Caesarean section were included in the study. Early onset PE was defined as hypertension (> 160/110 mmHg) plus mild proteinuria or mild hypertension plus severe proteinuria (≥ 5 g protein/24-hour urine collection) with delivery at < 34 weeks gestation [42]. Control patients were those pregnant women who underwent caesarean section when they suffered from malposition and premature rupture of membranes. None of the participants had any history of hypertension, diabetes, cardiovascular disease, kidney disease, hyperthyroidism, smoking, alcoholism, chemical dependency, intrauterine foetal death, foetal congenital or chromosomal abnormalities or pregnancies conceived by *in vitro* fertilization. Placental tissues were obtained from early onset severe preeclampsia pregnant women aged 20 to 35 years who underwent Caesarean deliveries between 2014 and 2016 in the Obstetrics and Gynaecology Hospital Affiliated to Nanjing Medical University of Jiangsu Province, China. All placental tissues were snap-frozen in liquid nitrogen immediately after Caesarean section. We used the distal edge of the placenta for consistency. Approximately 0.1 g placenta tissues were collected.

### Cell culture and treatment

Extravillous trophoblasts (EVTs) are responsible for invasion and maternal vascular remodelling [43]. HTR-8/SVneo cells were purchased from the Institute of Biochemistry and Cell Biology (Chinese Academy of Sciences, Shanghai, China), and these cells were derived from placental trophoblast. HTR-8/SVneo cells were maintained in RPMI1640 (WISET INC, Shanghai, China) medium supplemented with 8% heat-inactivated foetal bovine serum (FBS) (HyClone, Australia) and 2% penicillin/streptomycin in standard culture conditions. The cells were cultured at 37°C with 5% CO_2_. The cells were transiently transfected with lentiviruses seeding in the 6-well plates overnight. For stable cell lines, we treated HTR-8/SVneo cells with 2 μ g/ml puromycin after lentivirus infection of RPAIN. Lentiviruses overexpressing RPAIN lncRNA (purchased form GenePharma, Shanghai, China), C1q (purchased form GenePharma, Shanghai, China) or a negative control (purchased form GenePharma, Shanghai, China) were cultured using the GenePharma infection reagent according to the manufacturer's instructions. In all cases, cells were incubated for 72 hours at 37°C in a 5% CO_2_ incubator, and gene overexpression levels were assessed by qRT-PCR.

### RNA extraction

Total RNA was extracted from forty snap-frozen placenta tissues using TRIzol reagent (Ambion, Life Technologies, USA) according to the manufacturer's protocol. The amount and quality of RNA were evaluated using the One Drop OD-1000+ Spectrophotometer, and RNA integrity was assessed by standard denaturing agarose gel electrophoresis.

### Quantitative real-time PCR (qRT-PCR) and statistical methods

Total RNA was extracted from frozen placenta specimens using TRIzol reagent, and a PrimeScript RT reagent Kit (TaKaRa, Japan) was used for the synthesis of cDNA by adding 1 μg total RNA to the RT Reaction Mix. According to the manufacturer's instruction, the reverse transcription was performed at 37°C for 15 min and 85°C for 5 s. The qRT-PCR primers were designed using Primer3.0 and blasted specifically in NCBI. PCR was performed in a total reaction volume of 10 μl, including 5 μl SyBR Green, 0.5 μl of PCR forward primer, 0.5 μl of PCR reverse primer, 1 μl of cDNA, and 3 μl of RNase-free water. The qRT-PCR reaction was performed at an initial denaturation step of 10 min at 95°C followed by 40 cycles of 95°C for 15 s and 60°C for 1 min. All samples were normalized via the amplification of glyceraldehyde-3-phosphate dehydrogenase (GAPDH) RNA. The sequence of the primers was as follows: RPAIN (Forward: 5′-CGCTCCCTGTACAAACTGGT-3′, Reverse: 5′-GCCATAGTTTCATGGCAGGC-3′, C1q (Forward: 5′-AGAAGCGAAATTAGTGCGGAA-3′, Reverse: 5′-CCACGAAATTGGGAGTTGATGTC-3′ and GAPDH (Forward:5′-GACTCATGACCACAGTCCATGC-3′, Reverse: 5′-AGAGGCAGGGATGATGTTCTG-3′). ViiA7 real-time PCR System-Life Tech (Applied Biosystems, USA) was used to perform the qRT-PCR and data collection. Each experiment was performed in triplicate. The relative RNA level was calculated by the 2-ΔCT method, and ΔCT indicated the subtraction of the cycle threshold (CT) value for GAPDH from the CT value for the RNAs of interest.

### CCK-8 assay for cell proliferation

The proliferation capacity of the trophoblast cells was assessed by Cell Counting Kit-8 (CCK-8) (Vazyme Biotech Co., Ltd., Nanjing, China) assay according to the manufacturer's instructions. The HTR-8/SVneo cells were plated into the 96-well plate after being transfected with lentiviruses overexpressing RPAIN or a negative control. These cells were cultured for 6 h with five replicate wells at 2,000 cells per well. The cells were cultured at 37°C with 5% CO_2_. Then, 10 μl of the CCK-8 solution were added to the medium after the cells were incubated for 0, 24, 48, and 72 h. Finally, the absorbance was measured at 450 nm with a multifunctional microplate reader. Each experiment was performed in triplicate.

### *In vitro* cell invasion assay

The invasion capacity of the trophoblast cells was assessed by Transwell assay. One day prior to the experiment, 200 μg/ml Matrigel (BD Biosciences, USA) were added per well of a Transwell plate. HTR-8/SVneo cells were plated into the Transwell plate (8 μm PET, Millipore, Switzerland) after transfection with lentiviruses overexpressing RPAIN, a negative control or an empty vector. These cells were cultured in three replicate plates at 50,000 cells per plate. The cells were cultured at 37°C with 5% CO_2_. After 48 h of incubation, fixed with 4% paraformaldehyde for 30 minutes, stained with 0.1% crystal violet for 30 minutes. Finally, the number of invaded cells was examined using a digital microscopy at 100×. The invading cells were eluted with 100 μL of 1% RIPA (Beyotime, Shanghai, China). The absorbances were then measured at 575 nm using a multifunctional microplate reader. Each experiment was performed in triplicate.

### Flow cytometry (FCM)

HTR-8/SVneo cells were transiently transfected with lentiviruses for 7 2 h. The old-medium is collected and washed with PBS twice. Then, the cells were harvested using trypsin without EDTA; cells were double stained with Annexin V-PE/7-AAD Apoptosis Detection Kit (BD Biosciences, Shanghai, China) according to the manufacturer's instructions. The cells were sorted into survival, necrotic, early apoptotic, and late apoptotic cells. The relative ratio of apoptotic cells was assessed for further comparisons. The assay was performed in triplicate.

### Western blot analysis

HTR-8/SVneo cells transfected with overexpressing lentiviruses groups were lysed for protein extraction using mammalian reagent RIPA (Beyotime, Shanghai, China). The concentration of the protein samples was determined with the BCA Assay Kit (Beyotime, Shanghai, China). A total of 20 g proteins from each sample were separated by SDS-PAGE and transferred onto a PVDF membrane (Millipore, Bedford, MA, USA). The separated proteins were incubated with specific antibodies (MMP2, MMP9 and C1q, all purchased from Abcam) at 1:1000 concentration and specific antibodies (KI67, PCNA, Caspase-3 and BCL-2; Abcam) at 1:2000 concentration at 4°C overnight. The secondary antibody was horseradish peroxidase (HRP)-conjugated goat anti-mouse IgG at 1:2000 concentration. GAPDH was used as a loading control at 1:1000 concentration. All experiments were repeated at least thrice.

### Statistical analysis

We used the SPSS 20.0 software for data analysis. The data are expressed as the means ± standard deviations (SD) or Student's *t*-test. A value of *P* < 0.05 was considered statistically significant.
